# Prevalence of obesity and associated cardiovascular risk: the DARIOS study

**DOI:** 10.1186/1471-2458-13-542

**Published:** 2013-06-05

**Authors:** Francisco Javier Félix-Redondo, María Grau, José Miguel Baena-Díez, Irene R Dégano, Antonio Cabrera de León, Maria Jesús Guembe, María Teresa Alzamora, Tomás Vega-Alonso, Nicolás R Robles, Honorato Ortiz, Fernando Rigo, Eduardo Mayoral-Sanchez, Maria José Tormo, Antonio Segura-Fragoso, Daniel Fernández-Bergés

**Affiliations:** 1 Centro de Salud Villanueva Norte, Servicio Extremeño de Salud, Villanueva de la Serena, Badajoz, Spain; 2 Unidad de Investigación Grimex. Programa de Investigación en Enfermedades Cardiovasculares PERICLES, Villanueva de la Serena, Badajoz, Spain; 3 Grupo de Epidemiología y Genética Cardiovascular, Programa de Investigación en Procesos Inflamatorios y Cardiovasculares, IMIM (Institut Hospital del Mar d’Investigacions Mèdiques), Barcelona, Spain; 4 Centre d’Atenció Primària La Marina. Unitat de Recerca Barcelona Ciutat, Institut de Recerca en Atenció Primària Jordi Gol, Institut Català de la Salut, Barcelona, Spain; 5 Unidad de Investigación de Atención Primaria y del Hospital Universitario Señora de Candelaria. Medicina Preventiva y Salud Pública, Universidad de La Laguna, Santa Cruz de Tenerife, Spain; 6 Grupo de Investigación Riesgo Vascular en Navarra (RIVANA), Servicio de Investigación, Innovación y Formación Sanitaria, Departamento de Salud, Gobierno de Navarra, Pamplona, Spain; 7 Centro de Salud Riu Nord- Riu Sud, Santa Coloma de Gramenet, Barcelona; 8 USR Metropolitana Nord, IDIAP Jordi Gol, Mataró, Spain; 9 Dirección General de Salud Pública e Investigación, Desarrollo e Innovación, Consejería de Sanidad de la Junta de Castilla y León, Valladolid, Spain; 10 Hospital Universitario Infanta Cristina, Badajoz, Spain; 11 Servicio de Epidemiologia, Dirección General de Atención Primaria, Consejería de Sanidad Comunidad de Madrid, Madrid, Spain; 12 Grupo Cardiovascular de Baleares redIAPP, UB Génova. C. S. Sana Agustín, Palma de Mallorca, Baleares, Spain; 13 Plan Integral de Diabetes de Andalucía, Servicio Andaluz de Salud. CIBER de Fisiopatología de la Obesidad y Nutrición (CIBERobn), Instituto de Salud Carlos III, Madrid, Spain; 14 Servicio de Epidemiología, Consejería de Sanidad y Política Social de Murcia, Departamento de Ciencias Sociosanitarias, Universidad de Murcia.CIBER de Epidemiologia y Salud Pública (CIBERESP), Murcia, Spain; 15 Instituto de Ciencias de la Salud, Talavera de la Reina, Toledo, Spain; 16 Hospital Don Benito-Villanueva, Don Benito, Badajoz, Spain

**Keywords:** Cardiovascular diseases, Obesity, Prevalence, Risk factors

## Abstract

**Background:**

To estimate the prevalence of overweight and obesity in the Spanish population as measured with body mass index (BMI), waist circumference (WC) and waist to height ratio (WHtR) and to determine the associated cardiovascular risk factors.

**Methods:**

Pooled analysis with individual data from 11 studies conducted in the first decade of the 21st century. Participants aged 35–74 years were asked about the history of cardiovascular diseases, hypertension, diabetes and hypercholesterolemia. Height, weight, WC, blood pressure, glycaemia, total cholesterol, low-density and high-density lipoprotein cholesterol and coronary risk were measured. The prevalence of overweight (BMI 25–29.9 kg/m^2^), general obesity (BMI ≥30 kg/m^2^), suboptimal WC (≥ 80 cm and < 88 in women, ≥ 94 and < 102 in men), abdominal obesity (WC ≥88 cm ≥102 cm in women and men, respectively) and WHtR ≥0.5 was estimated, standardized for the European population.

**Results:**

We included 28,743 individuals. The prevalence of overweight and suboptimal WC was 51% and 30% in men and 36% and 22% in women, respectively; general obesity was 28% in both sexes and abdominal obesity 36% in men and 55% in women. The prevalence of WHtR ≥0.5 was 89% and 77% in men and women, respectively. All cardiovascular risk factors were significantly associated with abnormal increased values of BMI, WC and WHtR. Hypertension showed the strongest association with overweight [OR = 1.99 (95% confidence interval 1.81-2.21) and OR = 2.10 (1.91-2.31)]; suboptimal WC [OR = 1.78 (1.60-1.97) and OR = 1.45 (1.26-1.66)], with general obesity [OR = 4.50 (4.02-5.04), and OR = 5.20 (4.70-5.75)] and with WHtR ≥0.5 [OR = 2.94 (2.52-3.43), and OR = 3.02 (2.66-3.42)] in men and women respectively, besides abdominal obesity in men only [OR = 3.51 (3.18-3.88)]. Diabetes showed the strongest association with abdominal obesity in women [OR = 3,86 (3,09-4,89).

**Conclusions:**

The prevalence of obesity in Spain was high. Overweight, suboptimal WC, general, abdominal obesity and WHtR ≥0.5 was significantly associated with diabetes, hypertension, hypercholesterolemia and coronary risk. The use of lower cut-off points for both BMI and particularly WC and could help to better identify the population at risk and therefore achieve more effective preventive measures.

## Background

Cardiovascular diseases are the leading cause of death worldwide [1]. However, the decreasing trend observed in population cardiovascular mortality in recent years has been explained by changes in the prevention and control of cardiovascular risk factors and by the use of more effective medical and surgical treatments [2]. The increasing prevalence of obesity recently observed in Spain may have diluted to some extent the effect of other cardiovascular risk factors control to decrease coronary disease deaths [3-6].

The available evidence indicates that general obesity, measured with body mass index, and abdominal obesity, whether measured with waist circumference only or waist circumference corrected with height, are associated with coronary disease risk and mortality [7-10].

The prevalence of general and abdominal obesity has been recently studied in a national sample of Spanish general (>18 years) and elderly (≥65 years) population [11,12] and in regional samples [13-18]. However, the association of both types of obesity with the 10-year coronary disease risk estimated with the Framingham-REGICOR risk functions validated for the Spanish population [19] has not been studied in depth. The knowledge of this association may help to elucidate the excess risk presented by the population with overweight, general and abdominal obesity compared with the general population. This association may be particularly important in individuals aged 35 to 74 years, in whom the strategies of primary prevention of cardiovascular disease seem to be more effective [20].

The aims of this study are: (1) to estimate in the Spanish population aged 35 to 74 years the prevalence of overweight, general and abdominal obesity and (2) to estimate their association with cardiovascular risk factors and 10-year coronary disease risk as measured with the Framingham-REGICOR risk functions.

## Methods

### Study population

Pooled analysis with individual data from 11 population-based studies conducted in 10 geographical areas of Spain since 2000 with similar methodological designs. The methodology has been described elsewhere [21]. Briefly, participants were 35 to 74 years old and gave written informed consent to take part in the component studies. The DARIOS study (Dyslipemia, Atherosclerotic Risk, Increased high sensitivity C-reactive protein, and inflammatory and Oxidative status in Spanish population) was approved by the Municipal Healthcare Institute’s Clinical Research Ethics Committee (authorization nº 2009/3640).

### Anthropometric measurements

Waist circumference (WC), weight and height were measured with participants in underwear and barefoot. Body mass index (BMI) was determined as weight divided by squared height (kg/m^2^) and waist-to-height ratio (WHtR) as waist circumference (cm) divided by height (cm). All participants were classified according to BMI: (1) normal weight, BMI <25 kg/m^2^; (2) overweight, BMI 25–29.9 kg/m^2^; and (3) general obesity, BMI ≥30 kg/m^2^; according to WC: (1) optimal, WC < 94 cm in men and <80 cm in women(2) suboptimal, WC 94–102 cm in men and WC 80–88 cm in women; and (3) abdominal obesity, WC ≥102 cm in men and WC ≥88 cm in women [22,23]; and according to WHtR: (1) <0.5 and (2) ≥0.5 [24,25].

### Other measurements

Standardized questionnaires were used to collect sociodemographic and lifestyle variables, and the previous history of cardiovascular disease (coronary artery disease and stroke) and treatments for diabetes, hypertension and hypercholesterolemia. Current smoking was defined as actively smoking within the preceding year.

Prevalence of hypertension, diabetes and hypercholesterolemia was considered if the participant had been diagnosed, was treated for these disorders or presented with systolic blood pressure ≥140 mmHg or diastolic blood pressure ≥90 mmHg, glycaemia ≥ 126 mg/dl or total cholesterol ≥240 mg/dl, respectively.

Blood pressure was measured with a periodically calibrated sphygmomanometer. A cuff adapted to upper arm perimeter (young, adult, obese) was selected for each participant. Measurements were performed in a seated position after a 5-minute rest. Two measurements were taken and the mean value was recorded for the study.

Blood samples were taken following >10 h fast. Analysis was performed in local laboratories on fresh blood or aliquots of serum stored at −80°C in samples not previously thawed. Triglycerides, glucose, total and high-density lipoprotein (HDL) cholesterol were measured using standard methods. When triglycerides were <300 mg/dL, low density lipoprotein (LDL) cholesterol was calculated using the Friedewald formula. Analysis of concordance of lipid profile results using a reference laboratory was performed to correct the few deviations observed [21].

Cardiovascular risk in all participants aged 35 to 74 years with no history of cardiovascular disease was calculated with the REGICOR function adapted from the original Framingham function and validated for the Spanish population [19].

### Statistical analysis

The mean value of BMI, WC and WHtR and the prevalence of the corresponding categories were calculated by sex, standardized for the European age distribution [26] and accompanied by the 95% confidence interval.

We summarized the baseline characteristics in three groups based on categories of BMI (i.e. normal weight, overweight and general obesity), WC ( optimal, suboptimal and abdominal obesity) and WHtR (<0.5 and ≥0.5), using percentages for categorical data, means and standard deviations for normally distributed data, and median and interquartile range when the distribution departed from normal (e.g., glycaemia and triglycerides). We tested for differences and linear trend using Student *t* test, U-Mann Whitney and χ^2^ as appropriate.

To determine whether the associations found between BMI, WC and WHtR categories, and between cardiovascular risk factors and coronary risk were independent of age, we fitted multinomial logistic regression models adjusted for age.

Statistical analysis was done with R Statistical Package (R Foundation for Statistical Computing, Vienna, Austria; Version 2.15.0).

## Results

The study enrolled 28,887 participants from 11 epidemiological studies from 10 autonomous communities. Table 1 presents the mean BMI, WC and WHtR and the prevalence of general and abdominal obesity standardized to the European population. The prevalence in each component study of DARIOS is shown in Additional file 1: Tables S1 and S2.

**Table 1:** Mean and distribution by categories of body mass index and waist circumference for men and women, standardized to the European population

	**Men N = 13,425**	**Women N = 15,462**
BMI (kg/m^2^), mean (95% CI)	28.1 (28.0-28.1)	27.5 (27.5-27.6)
BMI categories, summarized,% (95% CI)		
Normal weight (<25)	21.3 (20.6-22.1)	36.1 (35.4-36.8)
Overweight (25–29.9)	50.7 (49.8-51.5)	35.6 (34.9-36.4)
General obesity (≥30)	28.0 (27.2-28.8)	28.3 (27.6-29)
Waist circumference (cm), mean	98.2 (98.0-98.4)	90.2 (90.0-90.4)
Waist circumference categories, summarized, % (95% CI)		
Men <94 cm; Women <80 cm	33.8 (32.9-34.7)	23.5 (22.8-24.2)
Men ≥94 and <102 cm; Women ≥80 and <88 cm	30.4 (29.6-31.3)	21.9 (21.2-22.6)
Men ≥102 cm; Women ≥88 cm	35.8 (34.9-36.7)	54.6 (53.8-55.4)
Waist-to-height ratio, mean	0.59 (0.58-0.60)	0.57 (0.57-0.58)
Waist-to-height ratio ≥0.5,% (95% CI)	89.1 (88.5-89.7)	77.3 (76.6-78.0)

ATPIII, Adult Treatment Panel III, *BMI* body mass index, *CI* confidence interval.

The prevalence of cardiovascular risk factors significantly increased with BMI category (normal weight, overweight, general obesity), except for smoking and HDL cholesterol, which significantly decreased in both men and women (Table 2). Similar results were found when we compared the prevalence of risk factors in different WC categories (Table 3) and WHtR (Additional file 1: Table S3).

**Table 2:** Population baseline characteristics by sex and body mass index category

**Men**	**Normal weight N = 2,760**	**Overweight N = 6,810**	**Obesity N = 3,801**	**p-value**	**p for linear trend**
Age, years, mean (SD)	51 (12)	54 (11)	55 (11)	<0.001	<0.001
Current smoker	1138 (41.2%)	2089 (30.7%)	1094 (28.8%)	<0.001	<0.001
Systolic blood pressure, mean (SD)	127 (18)	134 (18)	139 (18)	<0.001	<0.001
Diastolic blood pressure, mean (SD)	77 (10)	81 (10)	84 (11)	<0.001	<0.001
Hypertension	861 (31.4%)	3389 (50.0%)	2594 (68.4%)	<0.001	<0.001
Glycaemia, median [IQR]	93 [87–101]	98 [90–108]	103 [94–118]	<0.001	<0.001
Diabetes	239 (8.7%)	1066 (15.7%)	965 (25.5%)	<0.001	<0.001
Total cholesterol, mean (SD)	210 (38)	215 (39)	215 (40)	<0.001	<0.001
HDL cholesterol, mean (SD)	53 (12)	49 (11)	46 (10)	<0.001	<0.001
LDL cholesterol, mean (SD)	135 (34)	140 (35)	138 (34)	<0.001	0.004
Triglycerides, median [IQR]	96 [74–131]	114 [86–158]	134 [99–192]	<0.001	<0.001
Hypercholesterolemia	1016 (37.1%)	3204 (47.4%)	1975 (52.3%)	<0.001	<0.001
History of CV disease	121 (4.5%)	446 (6.8%)	338 (9.2%)	<0.001	<0.001
Waist circumference, mean (SD)	87 (7)	97 (7)	110 (9)	<0.001	<0.001
Waist-to-height ratio, mean (SD)	0.51 (0.05)	0.57 (0.04)	0.65 (0.05)	<0.001	<0.001
10-year CAD risk, median [IQR]	2.8 [1.5-5.2]	4.0 [2.3-7.1]	5.2 [3.0-8.3]	<0.001	<0.001
10-year CAD risk ≥10%	166 (6.6%)	716 (11.9%)	551 (16.7%)	<0.001	<0.001
**Women**	**Normal weight ** **N = 5,244**	**Overweight ** **N = 5,557**	**Obesity ** **N = 4,571**	**p-value**	**p for linear trend**
Age, years, mean (SD)	48 (10)	55 (11)	57 (10)	<0.001	<0.001
Current smoker	1546 (29.5%)	955 (17.2%)	481 (10.5%)	<0.001	<0.001
Systolic blood pressure, mean (SD)	117 (18)	128 (20)	137 (20)	<0.001	<0.001
Diastolic blood pressure, mean (SD)	73 (10)	78 (10)	82 (10)	<0.001	<0.001
Hypertension, (%)	1001 (19.2%)	2391 (43.3%)	3035 (66.6)	<0.001	<0.001
Glycaemia, median [IQR]	89 [83–95]	92 [86–101]	98 [90–111]	<0.001	<0.001
Diabetes	242 (4.6%)	572 (10.3%)	982 (21.6%)	<0.001	<0.001
Total cholesterol, mean (SD)	209 (38)	218 (39)	218 (38)	<0.001	<0.001
HDL cholesterol, mean (SD)	61 (13)	57 (13)	54 (12)	<0.001	<0.001
LDL cholesterol, mean (SD)	131 (34)	140 (34)	139 (34)	<0.001	<0.001
Triglycerides, median [IQR]	78 [62–101]	96 [73–128]	117 [88–157]	<0.001	<0.001
Hypercholesterolemia	1821 (35.0%)	2610 (47.3%)	2383 (52.5%)	<0.001	<0.001
History of CV disease	108 (2.1%)	211 (4.0%)	239 (5.4%)	<0.001	<0.001
Waist circumference, mean (SD)	79 (9)	90 (8)	104 (10)	<0.001	<0.001
Waist-to-height ratio, mean (SD)	0.49 (0.06)	0.58 (0.06)	0.67 (0.07)	<0.001	<0.001
10-year CAD risk, median [IQR]	1.2 [0.5-2.3]	2.4 [1.3-3.9]	3.5 [2.1-5.3]	<0.001	<0.001
10-year CAD risk ≥10%	24 (0.5%)	83 (1.6%)	185 (4.5%)	<0.001	<0.001

*CAD* Coronary artery disease, *CV* Cardiovascular, *HDL* High-density Lipoprotein, IQR Interquartile Range, *LDL* Low-density lipoprotein, *SD* Standard Deviation.

**Table 3:** Baseline characteristics by sex and categories of waist circumference

**Men**	**Waist circumference**				
	**<94 cm N = 3,702**	**≥94 and <102 cm N = 3,473**	**≥102 cm N = 4,215**	**p-value**	**p for linear trend**
Age, mean (SD)	50 (11)	54 (11)	56 (11)	<0.001	<0.001
Current smoker	1406 (38.0%)	1035 (29.8%)	1280 (30.4%)	<0.001	<0.001
Systolic blood pressure, mean (SD)	128 (17)	134 (18)	140 (18)	<0.001	<0.001
Diastolic blood pressure, mean (SD)	78 (10)	81 (10)	84 (11)	<0.001	<0.001
Hypertension	1219 (33.1%)	1764 (51.0%)	2894 (68.8%)	<0.001	<0.001
Glycaemia (mg/dl), median [IQR]	94 [88–102]	99 [91–109]	103 [93–117]	<0.001	<0.001
Diabetes	330 (8.9%)	539 (15.6%)	1038 (24.8%)	<0.001	<0.001
Total cholesterol (mg/dl), mean (SD)	212 (38)	217 (39)	214 (40)	<0.001	0.021
HDL cholesterol (mg/dl), mean (SD)	51 (12)	49 (10)	47 (10)	<0.001	<0.001
LDL cholesterol (mg/dl), mean (SD)	138 (34)	141 (34)	138 (34)	<0.001	0.884
Triglycerides (mg/dl), median [IQR]	102 [78–139]	120 [88–165]	132 [97–188]	<0.001	<0.001
Hypercholesterolemia	1438 (39.1%)	1710 (49.7%)	2181 (52.0%)	<0.001	<0.001
History of CV disease	150 (4.2%)	192 (5.8%)	402 (10.0%)	<0.001	<0.001
Body mass index (kg/m^2^), mean (SD)	24.7 (2.4)	27.7 (2.1)	31.7 (3.5)	<0.001	<0.001
Waist-to-height ratio, mean (SD)	0.51 (0.04)	0.58 (0.03)	0.65 (0.05)	<0.001	<0.001
10-year CAD risk, median [IQR]	2.8 [1.6-4.9]	4.1 [2.3-7.1]	5.4 [3.2-8.6]	<0.001	<0.001
10-year CAD risk ≥10%	184 (5.4%)	352 (11.4%)	650 (18.2%)	<0.001	<0.001
**Women**	**<80 cm N = 2,900**	**≥80 cm and <88 N = 2,800**	**≥88 cm N = 7,434**	**p-value**	
Age, mean (SD)	46 (9)	51 (10)	57 (11)	<0.001	<0.001
Current smoker	918 (31.7%)	671 (24.0%)	1019 (13.7%)	<0.001	<0.001
Systolic blood pressure, mean (SD)	115 (16)	122 (19)	134 (20)	<0.001	<0.001
Diastolic blood pressure, mean (SD)	72 (10)	75 (10)	80 (10)	<0.001	<0.001
Hypertension	465 (16.1%)	808 (29.0%)	4289 (57.9%)	<0.001	<0.001
Glycaemia (mg/dl), median [IQR]	88 [83–94]	90 [85–97]	96 [88–107]	<0.001	<0.001
Diabetes	87 (3.0%)	151 (5.4%)	1312 (17.8%)	<0.001	<0.001
Total cholesterol (mg/dl), mean (SD)	206 (36)	215 (37)	219 (38)	<0.001	<0.001
HDL cholesterol (mg/dl), mean (SD)	61 (13)	59 (12)	55 (12)	<0.001	<0.001
LDL cholesterol (mg/dl), mean (SD)	129 (32)	137 (33)	139 (34)	<0.001	<0.001
Triglycerides (mg/dl), median [IQR]	75 [60–95]	86 [68–114]	109 [82–149]	<0.001	<0.001
Hypercholesterolemia	881 (30.5%)	1163 (42.0%)	3807 (51.5%)	<0.001	<0.001
History of CV disease	69 (2.5%)	86 (3.2%)	326 (4.6%)	<0.001	<0.001
Body mass index (kg/m^2^), mean (SD)	22.7 (2.4)	25.3 (2.5)	30.9 (4.8)	<0.001	<0.001
Waist-to-height ratio, mean (SD)	0.46 (0.03)	0.53 (0.03)	0.64 (0.07)	<0.001	<0.001
10-year CAD risk, median [IQR]	0.9 [0.5-1.9]	1.7 [0.8-3.1]	3.1 [1.7-4.9]	<0.001	<0.001
10-year CAD risk ≥10%	9 (0.3%)	17 (0.7%)	229 (3.4%)	<0.001	<0.001

Abbreviation: *CAD* Coronary artery disease, *CV* Cardiovascular, *HDL* High-density Llipoprotein, *IQR* Interquartile range, *LDL* low-density lipoprotein, *SD* Standard deviation.

Overweight, suboptimal WC, general and abdominal obesity and high WHtR were associated with diabetes, hypertension, hypercholesterolemia and coronary risk independently of age in both sexes. The magnitude of these associations was higher in women than in men in all instances, except for hypercholesterolemia and the suboptimal WC category, which were higher in men (Tables 4, 5 and 6).

**Table 4:** Age-adjusted odds ratio of overweight and general obesity for cardiovascular risk factors by sex

	**Overweight (BMI ≥25 and <30)**				**General obesity (BMI ≥30)**			
	**Men**		**Women**		**Men**		**Women**	
	**OR (CI 95%)**	**p-value**	**OR (CI 95%)**	**p-value**	**OR (CI 95%)**	**p-value**	**OR (CI 95%)**	**p-value**
Age (1 year)	1.02 (1.02-1.03)	<0.001	1.06 (1.06-1.06)	<0.001	1.03 (1.03-1.04)	<0.001	1.09 (1.08-1.09)	<0.001
Diabetes	1.71 (1.47-1.99)	<0.001	2.53 (2.28-2.81)	<0.001	3.08 (2.64-3.60)	<0.001	2.95 (2.56-3.40)	<0.001
Hypertension	1.99 (1.81-2.21)	<0.001	2.10 (1.91-2.31)	<0.001	4.50 (4.02-5.04)	<0.001	5.20 (4.70-5.75)	<0.001
Hypercholesterolemia	1.45 (1.32-1.58)	<0.001	1.14 (1.05-1.24)	0.002	1.73 (1.57-1.92)	<0.001	1.22 (1.17-1.34)	<0.001
Coronary risk (1 percentage point)	1.11 (1.08-1.13)	<0.001	1.34 (1.30-1.39)	<0.001	1.18 (1.16-1.20)	<0.001	1.58 (1.53-1.63)	<0.001

Prevalence of hypertension, diabetes and hypercholesterolemia was considered if the participant was diagnosed, treated for these disorders or presented with systolic blood pressure ≥140 mmHg or diastolic blood pressure ≥90 mmHg, glycaemia ≥ 126 mg/dl or total cholesterol ≥240 mg/dl, respectively.

**Table 5:** Age-adjusted odds ratio of suboptimal waist circumference and abdominal obesity for cardiovascular risk factors by sex

	**Waist circumference**							
	**≥94 and <102 in men, and ≥80 and <88 in women**				**≥102 in men, and ≥88 in women**			
	**Men**		**Women**		**Men**		**Women**	
	**OR (CI 95%)**	**p-value**	**OR (CI 95%)**	**p-value**	**OR (CI 95%)**	**p-value**	**OR (CI 95%)**	**p-value**
Age (1 year)	1.03 (1.03-1.04)	<0.001	1.05 (1.04-1.06)	<0.001	1.05 (1.05-1.06)	<0.001	1.10 (1.10-1.11)	<0.001
Diabetes	1.54 (1.32-1.79)	<0.001	1.36 (1.04-1.80)	<0.001	2.48 (2.17-2.85)	<0.001	3.86 (3.09-4.89)	<0.001
Hypertension	1.78 (1.60-1.97)	<0.001	1.45 (1.26-1.66)	<0.001	3.51 (3.18-3.88)	<0.001	3.61 (3.21-4.07)	<0.001
Hypercholesterolemia	1.42 (1.29-1.56)	<0.001	1.22 (1.08-1.37)	<0.001	1.50 (1.37-1.65)	<0.001	1.30 (1.17-1.44)	<0.001
Coronary risk (1 percentage point)	1.10 (1.07-1.12)	<0.001	1.34 (1.27-1.42)	<0.001	1.17 (1.15-1.19)	<0.001	1.71 (1.62-1.79)	<0.001

Prevalence of hypertension, diabetes and hypercholesterolemia was considered if the participant has been already diagnosed, was treated for these disorders or presented with systolic blood pressure ≥140 mmHg or diastolic blood pressure ≥90 mmHg, glycaemia ≥ 126 mg/dl or total cholesterol ≥240 mg/dl, respectively.

**Table 6:** Age-adjusted odds ratio of waist-to-height ratio ≥0.5 for cardiovascular risk factors by sex

	**Waist-to-height ratio ≥0.5**			
	**Men**		**Women**	
	**OR (CI 95%)**	**p-value**	**OR (CI 95%)**	**p-value**
Age (1 year)	1.08 (1.07-1.09)	<0.001	1.10 (1.10-1.11)	<0.001
Diabetes	2.72 (2.04-3.62)	<0.001	2.73 (2.16-3.44)	<0.001
Hypertension	2.94 (2.52-3.43)	<0.001	3.02 (2.66-3.42)	<0.001
Hypercholesterolemia	1.81 (1.58-2.08)	<0.001	1.36 (1.23-1.51)	<0.001
Coronary risk (1 percentage point)	1.27 (1.22-1.32)	<0.001	1.68 (1.59-1.77)	<0.001

Reference value: Waist-to-height ratio <0.5. Model adjusted for age.

Prevalence of hypertension, diabetes and hypercholesterolemia was considered if the participant had been diagnosed, was treated for these disorders or presented with systolic blood pressure ≥140 mmHg or diastolic blood pressure ≥90 mmHg, glycaemia ≥ 126 mg/dl or total cholesterol ≥240 mg/dl, respectively.

**Figure 1:**
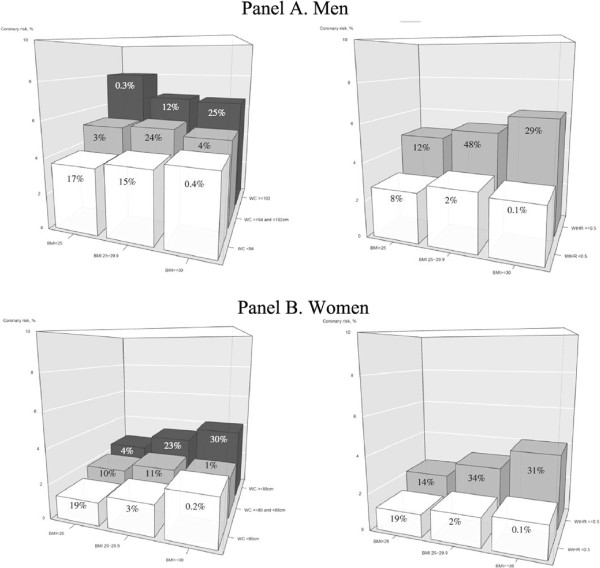
Coronary risk according to body mass index and waist circumference (left) and waist-to-height ratio (right) categories in men (Panel A) and in women (Panel B).

In men, coronary risk was directly related to increased BMI in the WC<94 category but increased in parallel with WC for all BMI categories. Notably, the highest mean coronary risk (7.6%) was identified in men with abdominal obesity and normal weight; however, only 0.2% of the sample presented this phenotype. In women, coronary risk increased with both WC and BMI. On the other hand, the risk of all individuals with WHtR ≥0.5 increased in parallel with BMI. Those with WHtR <0.5 presented minimal differences in coronary risk across BMI categories (Figure 1).

## Discussion

The present study showed that almost 80% and 65% of Spanish men and women, respectively, weigh more than the recommended values for their height. Indeed, 28% of men and women presented general obesity, 36% of men and 55% of women presented abdominal obesity, and 89% of men and 77% of women have a WHtR ≥0.5. Our results also showed a significant association independent of age between the obesity measures and cardiovascular risk factors (e.g., diabetes, hypertension and hypercholesterolemia). As a result, 10-year coronary disease risk significantly increased with the categories of BMI, WC and WHtR, which could indicate an important disease burden in coming years.

### Comparison with previous studies

The main Spanish study on cardiovascular risk factors (DORICA study) conducted between 1990 and 2000 in individuals aged 25–60 years reported a general obesity prevalence of 13% in men and 18% in women [27], lower than the DARIOS results in data collected since 2000. This supports the increasing trend in the prevalence of obesity evidenced in other studies [2-5]. On the other hand, the prevalence of general and abdominal obesity was lower in the nationwide ENRICA Study conducted between 2008 and 2010 than in DARIOS. The age ranges of the populations studied (>18 years in ENRICA and 35–74 years in DARIOS) may account for these differences [11]. However, since 2004 two studies have reported age-specific prevalence of general obesity in the Spanish elderly population [28,29] that is similar to DARIOS results. Since age is one of the main determinants of obesity, the prevalence is likely to increase as the population ages dramatically in coming years. The high prevalence of WHtR ≥0.5 observed could be related to the threshold chosen, which has been internationally recommended [25]. However, a higher threshold (0.55) better discriminated cardiovascular risk in a population with high prevalence of obesity [18].

An international study by Doak et al. showed that Romanian men and Bulgarian women aged 25–64 years presented the lowest prevalence of obesity among European countries (10%). On the contrary, Scottish men and women presented the highest prevalence (28% and 26%, respectively) [30], similar to the DARIOS results. Finally, analysis of National Health and Nutrition Examination Survey (NHANES) data showed that the prevalence in the US is around 32% [31]. The authors attributed the differences to the socioeconomic context of the countries studied [30].

### Cardiovascular risk factors, obesity and sex

Obesity is key in the development of hypertension and diabetes [32,33]. Indeed, both diseases were associated with general and abdominal obesity in the DARIOS data, independently of age. Hypercholesterolemia also showed a significant but weaker association, even though obese individuals in our sample presented the classical lipid disorder of hypertriglyceridemia and low HDL cholesterol [34].

In several population-based studies and a meta-analysis, the different measures of obesity were better discriminators and had a stronger association with cardiometabolic risk factors in women [10,18,24,25,35].

The sedentary life-style could be a possible cause, which is more prevalent in Spanish women than in men [36]. In addition, sex-related differences in fat distribution [37] and in eating behaviours [38] may play a key role. Further cohort studies are needed to ascertain sex-related differences in the use of these variables as predictors of cardiovascular events.

### Cardiovascular risk and obesity

A recent study has shown improved coronary risk prediction in men if a general obesity diagnosis is included [39], and higher mortality has been associated with overweight, general and abdominal obesity in men [40]. In DARIOS results, the baseline coronary risk was higher in men, although 10-year coronary disease risk was strongly associated with overweight, general and abdominal obesity in women as well. Previous studies in Spain report that obesity did not increase the incidence of cardiovascular events; however, further cohort studies with longer follow-up are needed [41,42]. In the Framingham Heart Study, for instance, obesity was associated with increased relative risk for development of cardiovascular disease in a population aged 35–75 and followed for 44 years [43].

### Coronary risk and obesity types

Finally, there is some controversy about the obesity measurement (i.e., general or abdominal) that better correlates to cardiovascular risk [18,24,25,35,44,45]. The abdominal obesity measures were significant predictors of cardiovascular events and death; BMI was not [10].

In our results, increased WC and WHtR implied higher coronary risk independently of BMI category. Surprisingly, men with WC ≥102 cm and BMI <25 kg/m^2^ presented the highest 10-year coronary disease risk. This finding could be explained by the sparse number of individuals included in this category. However, the subcutaneous fat storage in patients with high BMI seems to diminish cardiovascular risk compared to individuals with higher perivisceral fat storage [46]. Another possible explanation may be the presence of sarcopenic obesity (i.e., age-related body composition changes characterized by decreased skeletal muscle mass and increased body fat mass) [47] that is more associated with cardiometabolic risk [48] and mortality in individuals with coronary heart disease [49]. Both explanations may show the incapacity of the subcutaneous fat storage in these individuals due to genetics, ageing, sedentary lifestyle or unknown causes that result in ectopic fat storage with higher cardiometabolic risk [50,51]. In women, on the other hand, a risk gradient was found between BMI and both WC and WHtR. Therefore, we believe that both types of obesity should now be measured in the clinical setting.

### Strengths and limitations

The DARIOS Study includes 11 studies conducted in different regions of Spain in the first decade of the 21^st^ century. All these studies used standardized methodology. The DARIOS data is drawn from 10 Autonomous Communities that comprise approximately 70% of the total Spanish population aged 35–74 years. In addition, the sample size (>28,000 individuals) and response rate (>70% in 8 out of 11 studies) ensure that our results accurately reflect the prevalence of obesity in Spain. The response rate was estimated according to the cooperation rate in the 2011 guidelines of The American Association for Public Opinion Research [52].

The cross-sectional design of the study limits the causal interpretation of the associations described. Therefore, cohort studies are needed to ascertain the role of obesity in the incidence of coronary events, particularly in our society, where the prevalence of this cardiovascular risk factor has dramatically increased in recent years [3]. Notably, the cut-off point 0.55 for WHtR has shown higher predictive value for assessing the risk of diabetes and cardiovascular events [10,53].

## Conclusion

The prevalence of general and abdominal obesity in Spain was high: 28% of men and women presented weight values above those recommended for their height. On the other hand, the prevalence of increased WC was 36% and 55% in men and women, respectively. Diabetes, hypertension, hypercholesterolemia and 10-year coronary risk were significantly associated with all categories of general and abdominal obesity. Therefore, these lower cut-off points for both BMI and particularly WC could be used to identify the population at risk and effective preventive measures.

## Abbreviations

BMI: Body mass index; HDL: High-density lipoprotein; LDL: Low-density lipoprotein; WC: Waist circumference.

## Competing interests

The authors declare that they have no competing interests.

## Authors’ contributions

FJF participated in the design of the study and wrote the manuscript. MG performed the statistical analysis and participated in the manuscript writing. JMB participated in the design of the study and in the data collection. The other authors provided data and participated in the critical appraisal of the manuscript. All authors read and approved the final manuscript.

## Pre-publication history

The pre-publication history for this paper can be accessed here:

http://www.biomedcentral.com/1471-2458/13/542/prepub

## Supplementary Material

Additional file 1: Table S1 Anthropometric measurements in men, standardized to the European population. Table S2. Anthropometric measurements in women standardized to the European population. Table S3. Baseline characteristics by sex and categories of waist-to-height ratio. Click here for file
