# Supplementary material for: Prevalence of obesity and associated cardiovascular risk: the DARIOS study
Source: BMC Public Health. 2013 Jun 5;13:542. doi: 10.1186/1471-2458-13-542 (PMC3695785; doi:10.1186/1471-2458-13-542)
Supplement: Additional file 1: Table S1 — Anthropometric measurements in men, standardized to the European population. Table S2. Anthropometric measurements in women standardized to the European population. Table S3. Baseline characteristics by sex and categories of waist-to-height ratio. [file 1471-2458-13-542-S1.docx]

Prevalence of obesity and associated cardiovascular risk: the DARIOS Study

Francisco Javier Félix-Redondo^1,2^, María Grau^3*^, José Miguel Baena-Díez^1,3^, Irene R. Dégano^3^, Antonio Cabrera de León^5^, Maria Jesús Guembe^6^, María Teresa Alzamora^7,8^, Tomás Vega-Alonso^9^, Nicolás R. Robles^2,10^, Honorato Ortiz^11^, Fernando Rigo^12^, Eduardo Mayoral-Sanchez^13^, Maria José Tormo^14^, Antonio Segura-Fragoso^15^, Daniel Fernández-Bergés^2,16^

^1^Centro de Salud Villanueva Norte, Servicio Extremeño de Salud, Villanueva de la Serena, Badajoz, Spain

^2^Unidad de Investigación Grimex. Programa de Investigación en Enfermedades Cardiovasculares PERICLES, Villanueva de la Serena, Badajoz, Spain

^3^Grupo de Epidemiología y Genética Cardiovascular, Programa de Investigación en Procesos Inflamatorios y Cardiovasculares, IMIM (Institut Hospital del Mar d’Investigacions Mèdiques), Barcelona

^4^Centre d’Atenció Primària La Marina. Unitat de Recerca Barcelona Ciutat, Institut de Recerca en Atenció Primària Jordi Gol, Institut Català de la Salut, Barcelona, Spain

^5^Unidad de Investigación de Atención Primaria y del Hospital Universitario Señora de Candelaria. Medicina Preventiva y Salud Pública, Universidad de La Laguna. Santa Cruz de Tenerife, Spain

^6^Grupo de Investigación Riesgo Vascular en Navarra (RIVANA), Servicio de Investigación, Innovación y Formación Sanitaria, Departamento de Salud, Gobierno de Navarra. Pamplona, Spain

^7^Centro de Salud Riu Nord- Riu Sud. Santa Coloma de Gramenet, Barcelona

^8^USR Metropolitana Nord, IDIAP Jordi Gol, Mataró, Spain

^9^Dirección General de Salud Pública e Investigación, Desarrollo e Innovación, Consejería de Sanidad de la Junta de Castilla y León, Valladolid, Spain

^10^Hospital Universitario Infanta Cristina, Badajoz, Spain

^11^Servicio de Epidemiologia, Dirección General de Atención Primaria, Consejería de Sanidad Comunidad de Madrid. Madrid, Spain

^12^Grupo Cardiovascular de Baleares redIAPP, UB Génova. C. S. Sana Agustín, Palma de Mallorca, Baleares, Spain

^13^Plan Integral de Diabetes de Andalucía, Servicio Andaluz de Salud. CIBER de Fisiopatología de la Obesidad y Nutrición (CIBERobn), Instituto de Salud Carlos III, Madrid, Spain

^14^Servicio de Epidemiología, Consejería de Sanidad y Política Social de Murcia, Departamento de Ciencias Sociosanitarias, Universidad de Murcia.CIBER de Epidemiologia y Salud Pública (CIBERESP), Murcia, Spain

^15^Instituto de Ciencias de la Salud, Talavera de la Reina, Toledo, Spain

^16^Hospital Don Benito-Villanueva, Don Benito, Badajoz, Spain

Corresponding author:

Maria Grau, Cardiovascular Epidemiology and Genetics

IMIM (Hospital del Mar Medical Research Institute)

88 Dr Aiguader St, 08003 Barcelona, Spain

e-mail: mgrau@imim.es

Supplementary Table 1. Anthropometric measurements in men, standardized to the European population

| \|  \| ARTPER  N=1,493 \| CDC  N=2,054 \| CORSAIB  N=804 \| DINO  N=443 \| DRECA  N=736 \| HERMEX  N=1,046 \| PREDIMERC  N=966 \| RECCYL  N=1,198 \| REGICOR  N=2,685 \| RIVANA  N=1,765 \| TALAVERA  N=235 \| \| --- \| --- \| --- \| --- \| --- \| --- \| --- \| --- \| --- \| --- \| --- \| --- \| \| BMI, mean \| 28.7  (28.5-28.9) \| 28.3  (28.1-28.5) \| 27.6  (27.3-27.9) \| 28.1  (27.8-28.5) \| 28.7  (28.4-28.9) \| 29.2  (29-29.5) \| 28.0  (27.7-28.2) \| 27.9  (27.6-28.1) \| 27.6  (27.5-27.8) \| 27.6  (27.4-27.7) \| 28.3  (27.7-29) \| \| BMI categories \|  \|  \|  \|  \|  \|  \|  \|  \|  \|  \|  \| \| <18.5 \| 0.4  (0-0.7) \| 0.3  (0.1-0.6) \| 0.4  (0-0.9) \| 0.6  (0-1) \| 0  (0-0) \| 0.1  (0-0.3) \| 0.3  (0-0.6) \| 0.1  (0-0.3) \| 0.3  (0.1-0.5) \| 0.2  (0-0.4) \| 0.2  (0-0.6) \| \| 18.5-24.9 \| 17  (15-19) \| 20  (18-22) \| 27  (24-30) \| 16  (13-20) \| 16  (14-19) \| 14  (12-17) \| 22  (19-25) \| 24  (21-26) \| 23  (21-25) \| 23  (21-25) \| 20  (14-25) \| \| 25-26.9 \| 19  (17-21) \| 18  (16-20) \| 20  (17-22) \| 23  (19-27) \| 19  (17-22) \| 17  (14-19) \| 21  (18-24) \| 20  (18-23) \| 24  (22-25) \| 24  (22-26) \| 18  (13-24) \| \| 27-29.9 \| 30  (28-33) \| 30  (27-32) \| 29  (26-32) \| 33  (29-38) \| 30  (27-34) \| 31  (28-33) \| 31  (28-34) \| 30  (27-32) \| 30  (28-31) \| 30  (28-32) \| 32  (25-38) \| \| 30-34.9 \| 27  (25-30) \| 27  (25-29) \| 20  (17-22) \| 22  (18-26) \| 28  (25-31) \| 29  (26-32) \| 21  (19-24) \| 21  (19-24) \| 19  (18-21) \| 19  (17-21) \| 24  (18-30) \| \| 35-39.9 \| 6  (4-7) \| 4  (3-5) \| 4  (3-6) \| 4  (2-6) \| 5  (3-6) \| 7  (6-9) \| 4  (3-6) \| 4  (3-5) \| 4  (3-4) \| 3  (2-3) \| 6  (3-10) \| \| ≥40 \| 1  (0.5-2) \| 0.9  (0.4-1) \| 0.7  (0.1-1) \| 0.8  (0-2) \| 1  (0.4-2) \| 3  (2-4) \| 0.7  (0.2-1) \| 0.9  (0.3-2) \| 0.6  (0.3-0.9) \| 0.5  (0.2-0.8) \| 0.6  (0-2) \| \| BMI categories, summarized \| \| \| \| \| \| \| \| \| \| \| \| \| Normal weight  (<25 kg/m2) \| 17  (15-19) \| 20  (18-22) \| 27  (24-30) \| 17  (13-20) \| 16  (14-19) \| 15  (12-17) \| 22  (20-25) \| 24  (21-26) \| 23  (22-25) \| 23  (21-25) \| 20  (14-26) \| \| Overweight  (25-29.9 kg/m2) \| 49  (46-52) \| 48  (45-51) \| 48  (45-52) \| 56  (51-61) \| 50  (46-53) \| 47  (44-50) \| 52  (49-55) \| 50  (47-53) \| 53  (51-55) \| 55  (52-57) \| 50  (43-57) \| \| General obesity  (≥30 kg/m2) \| 34  (32-37) \| 32  (29-34) \| 25  (22-28) \| 27  (23-31) \| 34  (31-37) \| 38  (36-41) \| 26  (23-29) \| 26  (23-29) \| 23  (22-25) \| 22  (21-24) \| 30  (24-37) \| \| Waist circumference, mean \| 101  (100-102) \| 98  (98-99) \| 97  (96-98) \| 100  (99-101) \| 101  (100-102) \| 101  (100-101) \| 96  (95-96) \| 96  (96-97) \| 96  (96-97) \| 98  (97-98) \| 100  (98-102) \| \| Waist circumference categories, summarized \| \| \| \| \| \| \| \| \| \| \| \| \| <94cm \| 23  (21-26) \| 33  (31-36) \| 38  (35-42) \| 25  (21-30) \| 23  (20-26) \| 26  (24-29) \| 46  (42-49) \| 40  (38-43) \| 42  (39-46) \| 35  (32-37) \| 28  (21-34) \| \| ≥94 and <102 cm \| 32  (29-34) \| 30  (28-33) \| 29  (26-33) \| 33  (29-38) \| 31  (28-35) \| 32  (29-35) \| 28  (25-30) \| 29  (26-32) \| 27  (24-31) \| 32  (30-34) \| 27  (21-33) \| \| ≥102cm \| 45  (42-48) \| 37  (34-39) \| 32  (29-36) \| 41  (37-46) \| 46  (42-49) \| 42  (39-45) \| 27  (24-30) \| 31  (28-33) \| 30  (27-34) \| 34  (31-36) \| 45  (39-52) \| \| Waist-to-height ratio, mean \| 0.60  (0.60-0.61) \| 0.58  (0.57-0.58) \| 0.57  (0.57-0.58) \| 0.59  (0.59-0.60) \| 0.59  (0.59-0.60) \| 0.60  (0.59-0.60) \| 0.56  (0.56-0.56) \| 0.57  (0.57-0.58) \| 0.56  (0.56-0.57) \| 0.57  (0.57-0.58) \| 0.60  (0.60-0.61) \| \| Waist-to-height ratio ≥0.5 (%) \| 95  (94-96) \| 90  (89-91) \| 87  (84-89) \| 96  (94-98) \| 94  (92-95) \| 95  (93-96) \| 82  (80-84) \| 87  (85-89) \| 83  (81-86) \| 90  (88-91) \| 91  (87-95) \| |  |  |  |  |  |  |  |  |  |  |  |
| --- | --- | --- | --- | --- | --- | --- | --- | --- | --- | --- | --- | --- | --- | --- | --- | --- | --- | --- | --- | --- | --- | --- | --- | --- | --- | --- | --- | --- | --- | --- | --- | --- | --- | --- | --- | --- | --- | --- | --- | --- | --- | --- | --- | --- | --- | --- | --- | --- | --- | --- | --- | --- | --- | --- | --- | --- | --- | --- | --- | --- | --- | --- | --- | --- | --- | --- | --- | --- | --- | --- | --- | --- | --- | --- | --- | --- | --- | --- | --- | --- | --- | --- | --- | --- | --- | --- | --- | --- | --- | --- | --- | --- | --- | --- | --- | --- | --- | --- | --- | --- | --- | --- | --- | --- | --- | --- | --- | --- | --- | --- | --- | --- | --- | --- | --- | --- | --- | --- | --- | --- | --- | --- | --- | --- | --- | --- | --- | --- | --- | --- | --- | --- | --- | --- | --- | --- | --- | --- | --- | --- | --- | --- | --- | --- | --- | --- | --- | --- | --- | --- | --- | --- | --- | --- | --- | --- | --- | --- | --- | --- | --- | --- | --- | --- | --- | --- | --- | --- | --- | --- | --- | --- | --- | --- | --- | --- | --- | --- | --- | --- | --- | --- | --- | --- | --- | --- | --- | --- | --- | --- | --- | --- | --- | --- | --- | --- | --- | --- | --- | --- | --- | --- | --- | --- | --- | --- | --- | --- | --- | --- | --- | --- | --- | --- | --- | --- | --- | --- | --- | --- | --- | --- | --- | --- | --- | --- | --- | --- | --- | --- | --- | --- | --- | --- | --- | --- | --- | --- | --- | --- | --- | --- | --- | --- | --- | --- | --- | --- | --- | --- | --- | --- | --- | --- | --- | --- | --- | --- | --- | --- | --- | --- | --- |

Supplementary Table 2. Anthropometric measurements in women standardized to the European population

| \|  \| ARTPER  N=1.739 \| CDC  N=2.661 \| CORSAIB  N=865 \| DINO  N=502 \| DRECA  N=863 \| HERMEX  N=1.158 \| PREDIMERC  N=1.037 \| RECCYL  N=1.238 \| REGICOR  N=3.009 \| RIVANA  N=2.097 \| TALAVERA  N=293 \| \| --- \| --- \| --- \| --- \| --- \| --- \| --- \| --- \| --- \| --- \| --- \| --- \| \| BMI mean \| 29.4  (29.1-29.6) \| 28.6  (28.4-28.8) \| 27.3  (27.0-27.7) \| 28  (27.6-28.3) \| 28.7  (28.4-29.1) \| 28.4  (28.1-28.7) \| 27.0  (26.7-27.3) \| 27.4  (27.1-27.6) \| 27  (26-27) \| 26.3  (26.1-26.5) \| 28.1  (27.4-28.8) \| \| BMI categories \|  \|  \|  \|  \|  \|  \|  \|  \|  \|  \|  \| \| <18.5 \| 0.1  (0-0.2) \| 0.5  (0.2-0.7) \| 1  (0.3-2) \| 0.8  (0-2) \| 0.2  (0-0.5) \| 0.3  (0-0.7) \| 0.6  (0.1-1.2) \| 0.9  (0.3-1) \| 1  (0.7-2) \| 1  (0.6-2) \| 0.5  (0-1) \| \| 18.5-25 \| 19  (17-21) \| 26  (24-28) \| 39  (36-42) \| 28  (24-32) \| 28  (25-31) \| 30  (28-33) \| 39  (37-42) \| 36  (34-39) \| 44  (42-45) \| 46  (44-48) \| 31  (25-37) \| \| 25-27 \| 17  (16-19) \| 14  (13-16) \| 16  (13-18) \| 19  (16-23) \| 16  (13-18) \| 16  (14-18) \| 17  (15-19) \| 15  (13-17) \| 16  (14-17) \| 16  (14-17) \| 18  (13-22) \| \| 27-30 \| 24  (22-26) \| 24  (22-26) \| 17  (15-20) \| 22  (18-26) \| 21  (18-24) \| 19  (17-21) \| 20  (18-23) \| 20  (18-23) \| 18  (16-19) \| 17  (16-19) \| 23  (18-28) \| \| 30-35 \| 26  (24-28) \| 25  (22.9-26.9) \| 18  (16-21) \| 23  (19-26) \| 21  (18-24) \| 21  (19-23) \| 16  (14-18) \| 19  (17-21) \| 15  (14-16) \| 13  (12-15) \| 16  (12-20) \| \| 35-40 \| 10  (9-12) \| 9  (7-10) \| 7  (5-9) \| 5  (3-7) \| 11  (9-13) \| 9  (8-11) \| 5  (4-7) \| 7  (5-8) \| 5  (5-6) \| 5  (4-6) \| 8  (5-11) \| \| ≥40 \| 4  (3-5) \| 2  (2-3) \| 3  (2-4) \| 2  (0.8-3) \| 4  (3-5) \| 4  (3-5) \| 2  (0.8-2) \| 2  (1-3) \| 2  (1-2) \| 2  (1-2) \| 4  (2-7) \| \| BMI categories, summarized \| \| \| \| \| \| \| \| \| \| \| \| \| Normal weight  (<25 kg/m^2^) \| 19  (17-21) \| 26  (25-28) \| 40  (37-43) \| 29  (25-33) \| 28  (25-31) \| 31  (28-33) \| 40  (37-43) \| 37  (35-40) \| 45  (43-46) \| 47  (45-49) \| 32  (26-37) \| \| Overweight  (25-29.9 kg/m^2^) \| 41  (39-44) \| 38  (36-40) \| 33  (30-36) \| 41  (37-46) \| 37  (34-40) \| 35  (32-38) \| 37  (34-40) \| 35  (33-38) \| 33  (31-35) \| 33  (31-35) \| 41  (35-47) \| \| General obesity  (≥30 kg/m^2^) \| 40  (38-42) \| 36  (34-38) \| 27  (25-30) \| 30  (26-34) \| 35  (32-39) \| 35  (32-37) \| 23  (21-26) \| 28  (25-30) \| 22  (21-24) \| 20  (18-22) \| 28  (22-33) \| \| Waist circumference, mean \| 95  (94-95) \| 91  (90-91) \| 87  (86-87) \| 91  (90-92) \| 96  (95-97) \| 96  (95-96) \| 83  (83-84) \| 91  (91-92) \| 88  (87-89) \| 88  (87-88) \| 94  (93-96) \| \| Waist circumference categories, summarized \| \| \| \| \| \| \| \| \| \| \| \| \| <80cm \| 19  (17-21) \| 21  (19-23) \| 21  (18-24) \| 23  (20-27) \| 18  (15-20) \| 20  (18-23) \| 24  (21-27) \| 21  (19-24) \| 23  (20-26) \| 26  (24-27) \| 24  (19-30) \| \| ≥80 and <88cm \| 70  (68-72) \| 59  (57-61) \| 45  (41-48) \| 58  (55-62) \| 70  (67-73) \| 68  (66-71) \| 31  (28-34) \| 58  (55-60) \| 46  (43-50) \| 46  (44-48) \| 66  (60-72) \| \| ≥88cm \| 89  (88-91) \| 80  (78-82) \| 65  (62-68) \| 82  (79-85) \| 88  (85-90) \| 89  (87-91) \| 55  (52-58) \| 79  (77-81) \| 70  (67-73) \| 71  (69-73) \| 90  (86-94) \| \| Waist-to-height ratio, mean \| 0.61  (0.61-0.62) \| 0.58  (0.57-0.58) \| 0.55  (0.55-0.56) \| 0.58  (0.57-0.58) \| 0.61  (0.60-0.61) \| 0.61  (0.61-0.62) \| 0.53  (0.52-0.53) \| 0.59  (0.58-0.59) \| 0.55  (0.55-0.56) \| 0.56  (0.55-0.56) \| 0.60  (0.59-0.61) \| \| Waist-to-height ratio ≥0.5 (%) \| 92  (90-93) \| 80  (79-82) \| 68  (65-71) \| 81  (77-84) \| 87  (85-89) \| 89  (87-91) \| 58  (55-61) \| 80  (78-83) \| 69  (66-72) \| 72  (70-74) \| 91  (87-95) \| |  |  |  |  |  |  |  |  |  |  |  |
| --- | --- | --- | --- | --- | --- | --- | --- | --- | --- | --- | --- | --- | --- | --- | --- | --- | --- | --- | --- | --- | --- | --- | --- | --- | --- | --- | --- | --- | --- | --- | --- | --- | --- | --- | --- | --- | --- | --- | --- | --- | --- | --- | --- | --- | --- | --- | --- | --- | --- | --- | --- | --- | --- | --- | --- | --- | --- | --- | --- | --- | --- | --- | --- | --- | --- | --- | --- | --- | --- | --- | --- | --- | --- | --- | --- | --- | --- | --- | --- | --- | --- | --- | --- | --- | --- | --- | --- | --- | --- | --- | --- | --- | --- | --- | --- | --- | --- | --- | --- | --- | --- | --- | --- | --- | --- | --- | --- | --- | --- | --- | --- | --- | --- | --- | --- | --- | --- | --- | --- | --- | --- | --- | --- | --- | --- | --- | --- | --- | --- | --- | --- | --- | --- | --- | --- | --- | --- | --- | --- | --- | --- | --- | --- | --- | --- | --- | --- | --- | --- | --- | --- | --- | --- | --- | --- | --- | --- | --- | --- | --- | --- | --- | --- | --- | --- | --- | --- | --- | --- | --- | --- | --- | --- | --- | --- | --- | --- | --- | --- | --- | --- | --- | --- | --- | --- | --- | --- | --- | --- | --- | --- | --- | --- | --- | --- | --- | --- | --- | --- | --- | --- | --- | --- | --- | --- | --- | --- | --- | --- | --- | --- | --- | --- | --- | --- | --- | --- | --- | --- | --- | --- | --- | --- | --- | --- | --- | --- | --- | --- | --- | --- | --- | --- | --- | --- | --- | --- | --- | --- | --- | --- | --- | --- | --- | --- | --- | --- | --- | --- | --- | --- | --- | --- | --- | --- | --- | --- | --- | --- | --- | --- | --- | --- |

Supplementary Table 3. Baseline characteristics by sex and categories of waist-to-height ratio

| **Men** | **Waist-to-height ratio** | | |
| --- | --- | --- | --- |
|  | **<0.5**  **N=1,152** | **≥0.5**  **N=10,227** | **p-value** |
| Age, mean (SD) | 46 (10) | 54 (11) | <0.001 |
| Current smoker | 501 (43.5%) | 3213 (31.4%) | <0.001 |
| Systolic blood pressure, mean (SD) | 123 (15) | 136 (18) | <0.001 |
| Diastolic blood pressure, mean (SD) | 76 (9) | 82 (10) | <0.001 |
| Hypertension | 244 (21.3%) | 5628 (55.2%) | <0.001 |
| Glycaemia (mg/dl), median [IQR] | 92 [85-99] | 99 [91-110] | <0.001 |
| Diabetes | 53 (4.6%) | 1854 (18.2%) | <0.001 |
| Total cholesterol (mg/dl), mean (SD) | 207 (37) | 215 (39) | <0.001 |
| HDL cholesterol (mg/dl), mean (SD) | 53 (12) | 48 (11) | <0.001 |
| LDL cholesterol (mg/dl), mean (SD) | 133 (33) | 139 (34) | <0.001 |
| Triglycerides (mg/dl), median [IQR] | 92 [73-121] | 121 [89-170] | <0.001 |
| Hypercholesterolemia | 348 (30.4%) | 4980 (49.0%) | <0.001 |
| History of CV disease | 31 (2.7%) | 713 (7.3%) | <0.001 |
| Body mass index (kg/m^2^), mean (SD) | 23.1 (2.3) | 28.8 (3.7) | <0.001 |
| Waist circumference, mean (SD) | 81 (7) | 101 (10) | <0.001 |
| 10-year CAD, median [IQR] | 1.9 [1.2-3.4] | 4.3 [2.4-7.4] | <0.001 |
| 10-year CAD risk ≥10% | 28 (2.6%) | 1157 (12.9%) | <0.001 |
| **Women** | **<0.5**  **N=2,774** | **<0.5**  **N=10,341** | **p-value** |
| Age, mean (SD) | 45 (9) | 55 (11) | <0.001 |
| Current smoker | 901 (32.5%) | 1702 (16.5%) | <0.001 |
| Systolic blood pressure, mean (SD) | 114 (16) | 131 (20) | <0.001 |
| Diastolic blood pressure, mean (SD) | 72 (9) | 79 (11) | <0.001 |
| Hypertension | 382 (13.8%) | 5172 (50.2%) | <0.001 |
| Glycaemia (mg/dl), median [IQR] | 88 [82-93] | 94 [87-104] | <0.001 |
| Diabetes | 84 (3.0%) | 1460 (14.2%) | <0.001 |
| Total cholesterol (mg/dl), mean (SD) | 204 (36) | 218 (38) | <0.001 |
| HDL cholesterol (mg/dl), mean (SD) | 61 (12) | 56 (12) | <0.001 |
| LDL cholesterol (mg/dl), mean (SD) | 128 (32) | 139 (33) | <0.001 |
| Triglycerides (mg/dl), median [IQR] | 74 [60-94] | 102 [78-140] | <0.001 |
| Hypercholesterolemia | 770 (28.0%) | 5073 (49.4%) | <0.001 |
| History of CV disease | 62 (2.3%) | 418 (4.2%) | <0.001 |
| Body mass index (kg/m^2^), mean (SD) | 22.5 (2.3) | 29.4 (4.9) | <0.001 |
| Waist circumference, mean (SD) | 74 (5) | 95 (12) | <0.001 |
| 10-year CAD, median [IQR] | 0.8 [0.4-1.7] | 2.7 [1.4-4.4] | <0.001 |
| 10-year CAD risk ≥10% | 7 (0.3%) | 246 (2.6%) | <0.001 |

CAD, coronary artery disease; CV, Cardiovascular; HDL, high-density lipoprotein; IQR, interquartile range; LDL, low-density lipoprotein; SD, standard deviation
